# Influence of 24 h Simulated Altitude on Red Blood Cell Deformability and Hematological Parameters in Patients with Fontan Circulation

**DOI:** 10.3390/metabo12111025

**Published:** 2022-10-26

**Authors:** Julian Alexander Härtel, Nicole Müller, Johannes Breuer, Jens Jordan, Jens Tank, Janina Bros, Benedikt Seeger, Emily Zollmann, Wilhelm Bloch, Marijke Grau

**Affiliations:** 1 Department for Pediatric Cardiology, University Hospital Bonn, 53127 Bonn, Germany; 2 Institute of Aerospace Medicine, German Aerospace Center e.V. (DLR), 51147 Cologne, Germany; 3 Medical Faculty, University of Cologne, 50923 Cologne, Germany; 4 Department of Molecular and Cellular Sports Medicine, German Sport University Cologne, 50933 Cologne, Germany

**Keywords:** red blood cells, Fontan circulation, hypoxia, hemorheology, RBC deformability, congenital heart defect, altitude capability

## Abstract

Patients with Fontan circulation are particularly dependent on low pulmonary vascular resistance because their lungs are passively perfused. Hypoxia drives pulmonary vasoconstriction; thus, red blood cell (RBC) deformability and stability of hematological parameters might be of particular importance, because alterations during hypoxia might further influence circulation. This study aimed to measure respective parameters in patients with Fontan circulation exposed to normobaric hypoxia. A total of 18 patients with Fontan circulation (16 to 38 years) were exposed to normobaric hypoxia (15.2% ambient oxygen). Blood samples were taken in normoxia, after 24 h in hypoxia, and 60 min after return to normoxia. Blood count, RBC age distribution, EPO, RBC deformability, marker of RBC nitric oxide, oxidative state, and RBC ATP were measured. Hypoxia increased oxidative stress in RBC, but without affecting RBC deformability. RBC age distribution remained unaffected, although EPO concentrations increased, followed by a rise in reticulocyte count at an already high hematocrit. NO metabolism was not affected by hypoxia. Modest normobaric hypoxia for 24 h did not impair RBC deformability in patients with Fontan circulation; however, the oxidative system seemed to be stressed. Given the high baseline Hct in these patients, hypoxia-induced erythropoiesis could adversely affect rheology with more prolonged hypoxia exposure.

## 1. Introduction

The Fontan procedure was applied for the first time in the 1970s, and allowed newborns with only one anatomic and/or functional heart chamber to survive until adulthood [1]. Since then, the operation procedure has been modified several times, with decreasing mortality, currently at less than 5%, and improved long-term outcomes and reduced complications [2]. Nevertheless, for completion of the Fontan-circulation, most patients must undergo at least three operations in the first 3–5 years after birth to connect the superior and inferior vena cava to the pulmonary arteries without interconnection of a subpulmonary pump [1]. Due to the unique passive pulmonary blood flow, physicians and scientists must deal with special conditions, including low cardiac output, increased venous and pulmonary artery pressure, impaired exercise capacity, endothelial and lymphatic dysfunction, chronic hypoxemia, arrhythmic events, and systemic venous thrombi [2,3,4]. Unlike in healthy individuals, cardiac output is mainly controlled by pulmonary perfusion and not by the heart itself, as it can be increased by improving flow properties to and into the lungs [2]. The dependence on pulmonary perfusion becomes even more important during conditions of reduced ambient oxygen, such as airplane travel, where inner cabin pressure during long distance flights can correspond to altitude exposure of up to 2438 m above sea level (masl) [5]. Further to decreasing oxygen saturation, pulmonary blood flow in patients with Fontan circulation might be impaired, due to hypoxic vasoconstriction and systemic vasodilation, with potentially great impact on the systemic circulation [6]. Interestingly, previous studies by Garcia et al., Staempfli et al., Takken et al., and Müller et al. investigating hypoxic exposure for a maximum of 6 h, did not show more pronounced hypoxia-related impairments in cardiopulmonary capacity or cardiac output in patients with Fontan circulation, in comparison to healthy controls [7,8,9,10].

An underestimated factor in this context might be hemorheology, which is defined by hematocrit (Hct), red blood cell (RBC) deformability, RBC aggregation, and plasma viscosity [11]. Deformability is a unique property of RBC to maintain oxygen and nutrient supply in the microcirculation, as capillary diameters of about 5 µm are almost half the size of RBC diameters of 7 to 8 µm [12,13]. Deformability is defined by a reversible change of RBC shape under applied forces (shear stress), and depends on the specific interaction of the cytoskeletal proteins, such as ankyrin and spectrin, with the cell membrane [11]. The cytoskeleton is anchored in the cell membrane by proteins such as band 3 or adducin, and can slide in the lipid bilayer [12]. In addition, deformability depends on cytoplasmatic viscosity, which is mainly determined by mean corpuscular hemoglobin concentration (MCHC) and surface-to-volume ratio [11,14]. Furthermore, deformability changes during RBC senescence, and might further be influenced by different factors, such as nitric oxide (NO), RBC adenosine triphosphate (ATP), and reactive oxygen and nitrogen species (ROS/RNS). Nevertheless, these factors are still under discussion, as mechanisms leading to impaired or improved deformability are not fully understood [11,15,16,17,18,19,20].

In hypoxic conditions, RBC parameters might be influenced due to stimulation of erythropoietin (EPO) which stimulates erythropoiesis, and thus, increasing reticulocyte count and Hct. A review by Ploszczyca et al. demonstrated significant EPO increases after 24 h of moderate hypoxia exposure (2000–3100 masl) [21]. Nevertheless, exact EPO kinetics depend on time and frequency of altitude exposure, as well as overall altitude. Additionally, physiological responses to increased EPO levels are still a matter of debate because studies indicate either increased Hb, Hct, RBC, and reticulocyte count [22,23,24], or no changes in hematological parameters [25,26]. Furthermore, patients with Fontan circulation showed higher baseline EPO values compared to healthy controls, which might indicate different EPO kinetics [27].

However, increases in RBC and Hct might increase blood viscosity and, in turn, might lower pulmonary blood flow [28]. Hypoxia itself might affect RBC deformability because of reduced oxygen supply, but the impact might depend on the severity of the hypoxic conditions and the exact underlying mechanism has not yet been fully described [16,29,30,31,32].

Two previous studies investigating RBC rheologic parameters in patients with Fontan circulation indicated higher low-shear blood viscosity with unchanged RBC deformability compared to patients affected by other conditions [33], or likely higher deformability compared to patients with atrial septal defects or healthy control persons [27]. The latter study also investigated the effects of normobaric hypoxia with a maximum of 2 h exposure time, showing no differences in RBC deformability compared to normoxic conditions. Nevertheless, in all altitude studies involving patients with Fontan circulation, hypoxia exposure was too short or not sufficiently intense to expect hematological and/or hemorheological adaptation processes [28]. Additionally, while studies on hematological changes following 24 h hypoxic exposures in healthy subjects are well described, these data are lacking for patients with Fontan circulation. This information is of particular interest, as this condition better reflects long-distance flights and short stays at altitude.

Therefore, this study is the first to examine hematological parameters and RBC deformability in patients with Fontan circulation over 24 h in a normobaric altitude chamber with 15.2 % ambient oxygen (O_2_), simulating 2500 masl. Healthy controls were not investigated in the present study because physiological responses to hypoxic conditions are well described, and the simulated altitude seems to be safe for healthy subjects [6,16,34]. Instead, we aimed to establish a scientific rationale for recommendations regarding air travel and high-altitude exposure in this particular patient group.

The study adds important information on RBC regulation for this unique congenital heart defect. In addition, the study might help physicians to alert patients to potential dangers and risks during altitude exposure.

## 2. Materials and Methods

### 2.1. Study Population

We included 18 patients with Fontan circulation in the study. General patient characteristics are listed in Table 1.

Due to a stay in the altitude chamber for 24 h, the minimum age for participation was 16 years. In addition, patients with Fontan circulation had to fulfill the following inclusion criteria: (I) hemodynamic stable disease with NYHA class I; (II) peripherally measured oxygen saturation (SpO_2_) at sea level >90%; (III) age between 16 and 40 years.

Patients with (I) pacemakers, (II) failing of the Fontan circulation, (III) fenestration of the extracardiac conduit, (IV) medication with sildenafil or bosentan, (V) claustrophobia, depression or other mental disability, (VI) sleep disorders, (VII) acute infections, or (VIII) smokers were not eligible to participate in the study.

Before participation in the study, written informed consent was obtained from all subjects involved in the study.

The study protocol was approved by the local ethic committee of the University of Bonn (application number 054/20) and the ethics committee of the Aerztekammer Nordrhein (application number 2020046), and complied with the declaration of Helsinki. The study protocol was registered at the German Clinical Trials Register (DRKS) (application number DRKS00025989).

### 2.2. Study Procedure, Blood Sampling, and Processing

From April to May 2022, we exposed patients to normobaric hypoxia in an altitude module (envihab, DLR, Cologne, Germany) with 15.2 % ambient O_2_, simulating an altitude of 2500 masl. Total exposition time was 24 h. O_2_ was replaced by nitrogen (N_2_) at constant air pressure (≈1013 hPa).

Venous blood was collected from the cubital vein at three different timepoints, into sodium heparin vacutainers (Becton, Dickinson and Company, Franklin Lakes, JE, USA) for RBC measurements, and into EDTA S-Monovetten^®^ (Sarstedt AG & Co. KG, Nürnbrecht, Germany) for differential blood count: In normoxia, before entering the normobaric altitude room (T1); in hypoxia, 24 h after hypoxia exposure within the hypoxic module (T2); and in normoxia, 60 min after leaving the normobaric altitude room (T3). In addition, SpO_2_ was measured at the indicated timepoints, using a finger pulse oximeter (IntelliVue X3, Philips, Amsterdam, The Netherlands). Processing of the blood samples was carried out in normoxia.

Unless otherwise described, the following parameters were examined at every timepoint: (1) blood count, (2) reticulocyte count (T1 + T2), (3) RBC age distribution, (4) plasma EPO concentrations, (5) RBC deformability, (6) RBC nitrite/RSNO/Fe-NO, (7) phosphorylation of RBC NOS at serine 1177 residue, (8) oxidative state, and (9) RBC ATP concentration.

Reticulocyte count was measured at the central laboratory of the University Hospital Bonn, whereas heparinized blood was processed immediately after sampling at the Laboratory of the DLR, and was either stored at appropriate temperature until final measurement or transported to the Department of Molecular and Cellular Sports Medicine at the German Sport University Cologne for immediate analysis. Details are described below.

Immediately after blood sampling, whole blood was mixed with 4% formaldehyde for the immunostaining of RBC-NOS phosphorylation site serine 1177 (see below). The remaining blood was centrifugated at 760 g for 15 min, and the supernatant was transferred into clean tubes and immediately stored at −80 °C for the measurement of EPO concentration.

The remaining RBC pellet was processed as described in the following sections.

### 2.3. Density Gradient Centrifugation

RBC were separated according to their cell density to yield three distinct fractions which were previously defined as young RBC (less dense, high deformability), old RBC (highest density, lowest deformability), and middle fraction (RBC between young and old RBC) [35,36,37]. Briefly, six Percoll solutions were prepared using a Percoll stock solution (1.130 g/mL; VWR International GmbH, Darmstadt/Germany) and SAH buffer (containing 26.3 g/L BSA, 132 mmol/L NaCl, 4.6 mmol/L and 1 mmol/L HEPES; pH 7.1) as dilution buffer. Two ml of each dilution was carefully transferred into a clean 15 mL, starting with the highest density.

RBC were washed in a nine-fold volume of isotonic GASP buffer (albumin and glucose: 9 mmol/L, Na2HPO4,1.3 mmol/L NaH2PO4, 140 mmol/L NaCl, 5.5 mmol/L glucose, 0.8 g/L BSA, pH 7.4), centrifugated, and diluted 1:1 with SAH. An amount 600 µL of this solution was layered on top of the gradients and centrifuged for 25 min at 3000 U/min. The top layer (young RBC), the bottom layer (old RBC), and all layers in between (main fraction) were separately transferred to respective tubes and washed with GASP buffer. After centrifugation (3500 g for 1 min), the supernatant was removed, and the RBC were first weighed to calculate the percent distribution of each RBC sub-fraction and then used for deformability measurements, as described above.

### 2.4. Red Blood Cell Deformability

RBC deformability of each sub-fraction was measured by laser diffraction analysis using a Laser-assisted Optical Rotational Red Cell Analyzer (LORRCA MaxSis, RR Mechatronics, Zwaag, The Netherlands), as described elsewhere [38,39,40].

Briefly, for laser diffraction analysis, 100 × 10^6^ RBC of each subfraction was mixed with a viscous polyvinylpyrrolidone solution (PVP, viscosity: 29 cP; RR Mechatronics, Zwaag, The Netherlands) in a 1:250 ratio. The necessary volume had been calculated from a blood count that was performed from the RBC pellet using an automatic cell analyzer, Sysmex Digitana KX-21N (Sysmex, Horgen, Switzerland). The RBC/PVP samples were inserted in a Couette flow system and were exposed to nine increasing shear stresses between 0.3 and 30 Pa. A laser beam was directed through the samples and was diverted by the RBC. Thus, increasing shear stresses changed the shape of the RBC and, as a consequence, affected the diffraction pattern of the laser beam which was analyzed by the LORRCA software and resulted in the calculation of an elongation index (EI) for each shear stress [15]. Maximum deformability at infinite shear stress and SS_1/2_, the shear stress at one-half EI_max_, was calculated by the LORRCA software and used to calculate the SS_1/2_:EI_max_ ratio. It should be mentioned that a reduced SS_1/2_:EI_max_ ratio represents increased RBC deformability. To calculate total deformability, the percentage share of each subfraction was multiplicated by a measured SS_1/2_:EI_max_ ratio of each sub-fraction, and the results of each sub-fraction was summarized.

For osmotic gradient ektacytometry (osmoscan), 1000 × 10^6^ RBC were transferred to 5 mL of PVP solution. Again, RBC volume was calculated from the blood count, as described above [38]. Osmoscan measurements were performed using a LORRCA MaxSis at constant shear stress and varying osmolality from 0–500 mOsmol/kg, as recently described [38]. The deformability values were automatically generated and included: O_min_, representing the osmolality at minimum deformability; EI_max_, reflecting the maximum deformability of RBC in isotonic solution; and O_hyper_, representing the hyperosmotic osmolality corresponding to 50% of EI_max_ [41].

### 2.5. RBC Nitrite/RSNO/Fe-NO

RBC nitrite/RSNO/Fe-NO concentration was measured using an ozone-based chemiluminescence NO detector (CLD 88e, EcoPhysics AG, Duernten, Switzerland) according to previously published protocols [42,43].

First, after centrifugation (see Section 2.2), the RBC pellet was immediately mixed with a nitrite preservation solution (0.8 M ferricyanide, 0.1 M N-ethylmaleimide, and 10 V-% Igepal), mixed, and stored at −80 °C until measurements were taken. Upon measurement, samples were thawed on ice and mixed with ice-cold methanol (VWR International GmbH, Darmstadt, Germany) in a 1:2 ratio and centrifuged at 3600 g, 4 °C for a total of 15 min. The supernatant was then collected into clean tubes and the nitrite/RSNO/Fe-NO concentration was measured by injecting 100 µL into an acidified tri-iodide solution, reducing nitrite to NO gas [35,44], which was analyzed by its gas-phase chemiluminescent reaction with ozone [44]. Data analysis was performed with Chart FIA software (eDAQ Chart v.5.5.11, Ecophysics, Switzerland) to integrate the area under the curve. A standard curve was used to calculate nitrite/RSNO/Fe-NO within the samples after correction of the concentration for nitrite/RSNO/Fe-NO levels of the used methanol and preservation solution. Each sample was measured three times.

### 2.6. Immunostaining of RBC NOS Serine 1177 Residue and Nitrotyrosine

Formaldehyde-fixed RBC were washed and dispersed on a slide. Immunostaining was precisely performed as previously published [45]. Each slide contained a test and control area. Samples were washed using tris-buffered saline (TBS), and the RBC membrane was permeabilized using 0.1% trypsin. Exogenous peroxidase was applied to block unspecific binding sites prior to incubation of the samples with the primary antibody (either Rabbit anti Human NOS III Serine 1177; 1:500; Merck, Darmstadt, Germany or Rabbit anti nitrotyrosine 1:500; Upstate/Millipore, Darmstadt, Germany). The control area was incubated without the primary antibody. Slides were washed using TBS, and both the control and the test area were incubated with the secondary antibody (goat anti-rabbit antibody; 1:150; Dako, Glostrup, Denmark). The staining was developed using 3,3-diaminobenzidine-tetrahydrochloride solution (Sigma-Aldrich, St. Louis, MI, USA), dehydrated in increasing alcohol solutions, mounted using Entellan^®^ (Merck, Darmstadt, Germany), and covered. Images of the stained RBC were taken using an Axiophot 1 microscope (Zeiss, Oberkochen, Germany) coupled to a camera (Progres Gryphax Prokyon; Jenoptik Optical Systems GmbH, Jena, Germany) with a 200-fold magnification. Stainings were analyzed with software ImageJ 1.52a (National Institutes of Health, Bethesda, MA, USA). For staining intensity analysis, mean gray values were measured, and total immunostaining intensity was calculated as previously described [46]

### 2.7. Oxidative Status

A standard number of 1 × 10^7^ RBC were used to analyze the ROS/RNS content using a commercial OxiSelect In Vitro ROS/RNSAssay Kit (Cell Biolabs Inc., San Diego, CA, USA), as previously described [16]. ROS/RNS within the samples reacts with dichlorodihydrofluorescin (DCFH), which is rapidly oxidized to the highly fluorescent 20,70-dichlorodihydrofluorescein form. The prepared dichlorodihydrofluorescin probe was added to the lysed RBC and standards, and the reaction was allowed to proceed for 45 min at room temperature. Fluorescence was read with a fluorescence plate reader (Fluoroskan Ascent Microplate Fluorometer; Thermo Fisher Scientific, Waltham, MA, USA) at 480 nm excitation and 530 nm emission. Samples were then measured against a dichlorodihydrofluorescin standard, and concentrations were calculated by linear regression.

Total antioxidant capacity (TAC) was measured using a respective Assay Kit (abcam, Berlin, Germany); 2 × 10^6^ RBC were used for this assay and processed as described by the manufacturer. During the measurement, a Cu^2+^ ion is converted to Cu^+^ by antioxidants within the sample. The Cu^+^ was chelated with a colorimetric probe, producing an absorbance peak at 570 nm, which was proportional to the total antioxidant capacity and was read using a Multiskan FC Photometer (Thermo Fisher Scientific, Waltham, MA, USA).

### 2.8. ATP and EPO Concentrations

An enzyme-linked immunosorbent assay Human EPO ELISA (R&D Systems, Minneapolis, MI, USA) was used to determine plasma EPO concentration according to the manufacturers’ instructions.

RBC ATP was measured in a standardized number of 1 × 10^6^ RBC using an ATP Assay Kit (Sigma-Aldrich, St. Louis, MI, USA). The RBC ATP concentration was determined by phosphorylating glycerol, resulting in a colorimetric (570 nm) product proportional to the amount of ATP present. The absorbance was read using a Multiskan FC Photometer (Thermo Fisher Scientific, Waltham, MA, USA).

### 2.9. Statistical Analysis

Statistical analysis was performed using GraphPad Prism (V. 9.4.0, GraphPad Software, La Jolla, CA, USA).

Only complete data sets in normoxia and hypoxia were included. As some blood samples had already been coagulated at the time of measurement, eleven data sets were analyzed for reticulocyte count.

D’agostino-Pearson normality test was performed to test data for normal distribution. Variables fitting a normal distribution were then tested with repeated measures of analysis of variance (ANOVA) to detect differences between timepoints. Post-hoc test was performed using Tukey’s multiple comparison test. For variables not fitting normal distribution, Friedman test was applied followed by Dunn’s multiple comparison test for post-hoc analysis.

Student’s paired *t*-test was used to detect differences between reticulocyte count measured at the laboratory of the University Hospital Bonn, as it was only measured at T1 and T2.

Deformability parameter analysis (O_min_, O_hyper_, Osmoscan EI_max_) was conducted using mixed effects analysis (normal distributed) or Kruskal Wallis test (not fitting normal distribution) due to different sample counts.

Quantitative data were presented as mean ± standard deviation (SD) unless otherwise described.

Significance level was defined as *p* ≤ 0.05 and different significance levels were demonstrated as follows: * *p* ≤ 0.05, ** *p* ≤ 0.01, *** *p* ≤ 0.001, **** *p* ≤ 0.0001, ns = not significant.

## 3. Results

### 3.1. Oxygen Saturation and RBC Parameters

SpO_2_ was significantly decreased after 24 h in hypoxia, but returned to initial values at T3 (60 min after leaving hypoxia), which showed no differences to values at T1 (Table 2). Reticulocyte count significantly increased 24 h after hypoxia exposition. Plasma EPO concentrations showed significantly increased values at T2 and T3, respectively, compared to T1 (Table 2). Neither RBC count nor Hb or Hct showed significant differences throughout the study (Table 2). However, baseline Hb and Hct were already higher than the standard values.

RBC indices (mean corpuscular volume (MCV), mean corpuscular hemoglobin (MCH), and mean corpuscular hemoglobin concentration (MCHC)) remained unaffected throughout the study.

pH and red cell distribution width (RDW) showed statistically significant increased values at T2 and T3 (Table 2). RBC ATP concentration did not change throughout the study (Table 2).

### 3.2. RBC Distribution of RBC Sub-Fractions and RBC Deformability

**Figure 1:**
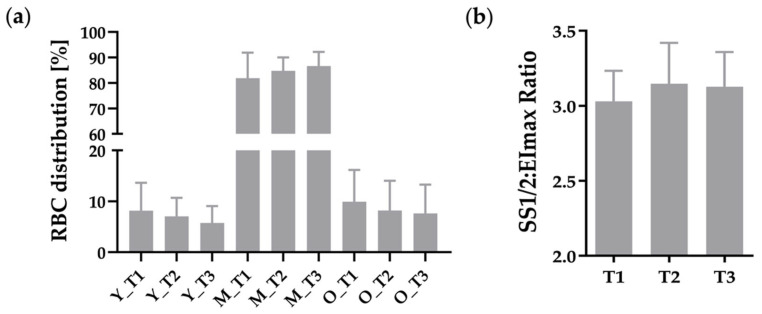
(**a**) Distribution of RBC subfractions in % with n = 11 at measured timepoints (T1, T2, and T3), indicating no significant changes due to hypoxia, Y = young RBC fraction, M = middle RBC fraction, O = old RBC fraction; (**b**) RBC deformability shown as SS_1/2_:EI_max_ ratio, demonstrating no influence by hypoxia.

Distribution of the different RBC sub-fractions young, middle, and old RBC are presented in Figure 1a, indicating no significant differences between the timepoints. Calculated combined RBC deformability did not change under hypoxic condition (Figure 1b).

**Figure 2:**
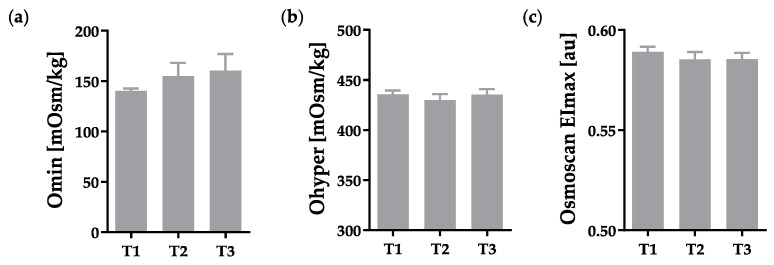
Deformability parameters under an osmotic gradient measured by Osmoscan, indicating no changes due to hypoxia with unaffected (**a**) O_min_, (**b**) O_hyper_, and (**c**) EI_max_. Datasets included in this figure: (**a**–**c**) T1: n = 14, T2: n = 11, T3: n = 8.

Deformability parameters under an osmotic gradient, obtained during Osmoscan, remained unaltered (Figure 2).

### 3.3. RBC-NOS Serine 1177 and RBC Nitrite/RSNO/Fe-NO

**Figure 3:**
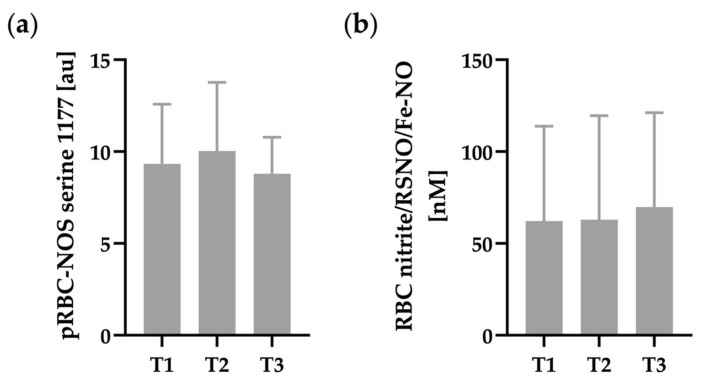
(**a**) Phosphorylation of RBC-NOS at serine 1177 residue and (**b**) corresponding RBC nitrite/RSNP/Fe-NO concentration were not affected by hypoxia. (**a**) n = 16 and (**b**) n = 17.

Staining intensity of RBC-NOS serine 1177 phosphorylation (Figure 3a) and RBC nitrite/RSNO/Fe-NO concentration (Figure 3b) remained unaffected by hypoxia exposure.

### 3.4. Oxidative Status of RBC

**Figure 4:**
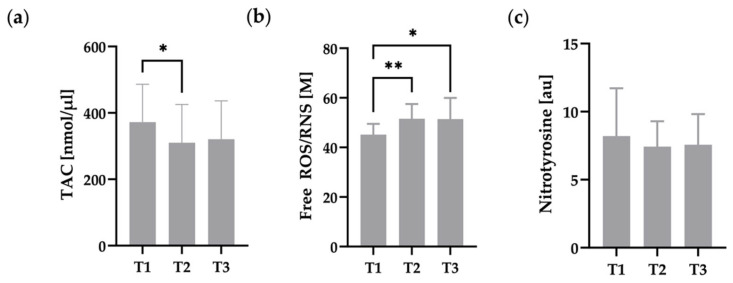
Oxidative status of RBC, indicating (**a**) significantly reduced total antioxidant capacity (TAC) and (**b**) significantly increased free ROS/RNS due to hypoxia. (**c**) Nitrotyrosine concentration remained unaffected during the study. (**a**–**c**) n = 16. * *p* ≤ 0.05, ** *p* ≤ 0.01.

Free radical content, TAC, and RBC nitrotyrosine are presented in Figure 4. Free ROS/RNS significantly increased from T1 to T2 and T3, respectively (Figure 4b). TAC was significantly decreased at T2 compared to T1. T3 still showed a trend for reduced TAC compared to T1. Nitrotyrosine, as marker for cellular damage, remained unaltered during the intervention.

## 4. Discussion

In recent decades, operation procedures for palliation of univentricular hearts have improved, with better outcomes for patients [47]. Therefore, current studies focus on improving quality of life in these patients [48,49,50]. Currently, exposure to hypoxia, i.e., during air travel, is part of daily life. Nevertheless, there is limited evidence regarding the potential risks for patients with Fontan circulation exposed to altitude, resulting in uncertainties for patients, family members, and physicians. These risks and uncertainties might even start at moderate altitudes of 2.500 masl or lower, which are common altitudes during long-distance flights and travel destinations, and are well tolerated by healthy individuals.

As patients with Fontan circulation only have a passive pulmonary perfusion, because a subpulmonary ventricle is missing, RBC deformability, as important factor of blood viscosity, and other hematological parameters might be relevant factors in maintaining blood circulation and oxygen supply, especially in hypoxia due to hypoxic pulmonary vasoconstriction.

Our study is the first to demonstrate the impact of a 24-h hypoxia exposure on RBC deformability and hematological parameters in patients with Fontan circulation. Since previous studies have not yet exceeded a maximum hypoxia exposure time of 6 h [7,8,27], this study offers new insights into this unique and complex heart defect in artificial altitude. The key findings of the present study are that RBC deformability is not affected by 24-h hypoxia exposure in patients with Fontan circulation despite increased free radical content and reduced TAC, suggesting an adequate antioxidant capacity in RBC, at least during the study period. In addition, NO metabolism, as well as ATP concentration in RBC, were not affected by the applied hypoxia stimulus. However, we observed increased EPO and reticulocyte count 24 h after hypoxia exposure.

Reduced oxygen saturation is the driving force for hypoxia adaptation. In patients with Fontan circulation, SpO_2_ is already diminished in normoxia, likely due to returning blood from the coronary sinus, which drains in the atrium and probably veno-venous collaterals [2]. With a SpO_2_ of 94 ± 3%, our patients showed remarkably good values. The findings might be explained by a selection bias, as patients had to have a SpO_2_ ≥ 90 % to participate in the study. Hypoxia exposure reduced SpO_2_ values, and levels were lower than described in the study by Takken et al. [8], but similar to those in the study by Müller et al. [10]. A loss of 7.4% of SpO_2_ between normoxia and hypoxia was higher compared to healthy controls in the studies by Takken et al. and Müller et al., under similar altitude conditions (loss of 4.1% respective 5.1%) [8,10], which might be explained by the relative increase of shunt perfusion due to hypoxic pulmonary vasoconstriction, and which might indicate that hypoxia conditions result in a more pronounced response in patients with Fontan circulation than in healthy controls. However, 24-h hypoxia exposure might not lead to more increased hypoxemia than shorter exposition times. In addition, we observed that SpO_2_ immediately recovered after leaving the altitude chamber, showing no difference to pre-intervention levels after 60 min in normoxia.

Our patients showed increased RBC counts, Hct, and Hb at baseline than described normal values, which might be related to stimulated erythropoiesis caused by increasing EPO concentrations, in general as a result of reduced SpO_2_, as previously demonstrated [27,51]. Elevated Hct values are commonly associated with increased blood viscosity [11], which might impair pulmonary blood flow and the cardiopulmonary circulation. Long stays at altitudes > 2500 masl are reported to further increase Hct [34], which is why blood viscosity might become even more pronounced in patients with Fontan circulation. Indeed, blood viscosity is an often neglected contributor to vascular resistance [33]. We speculate that increased blood viscosity might be an additional factor for reduced cardiopulmonary capacity, as demonstrated for these patients [52]. The data presented herein did not provide evidence of higher Hct values 24 h after hypoxia exposure. These results are in line with a recent study by Breda et al., which showed no difference in hematological parameters during the first days at 2500 masl in subjects with a healthy heart [34].

While approximately 2 h of altitude exposure did not affect EPO levels in patients with Fontan circulation or healthy controls [27], we observed a significant two-fold increase in plasma EPO concentration after 24 h of hypoxia. Doubling of EPO levels after 24 h in hypoxia is also described for healthy individuals [21,28,53]. This might indicate that the hypoxia stimulus results in comparable physiological reactions in both healthy subjects and patients with Fontan circulation. Yet, given the higher baseline EPO levels in patients with Fontan circulation [27], one might speculate that the additional EPO increase might result in higher Hct once the newly formed RBC are released into circulation. Since this might affect blood viscosity, as described above, it may be prudent to pay attention to sufficient fluid intake in patients with Fontan circulation exposed to hypoxia. As water vapor pressure is reduced and humidity is low in hypobaric hypoxia, there is increased water loss through ventilation, resulting in a further increase of Hct [54,55]. In addition, diuresis is increased due to increased bicarbonate excretion and hemoconcentration during hypoxic adaptation [54,56]. These mechanisms could be even more relevant during physical activity in hypoxia, as sweating increases and further exacerbates fluid loss. Furthermore, increased EPO levels might be related to higher RBC turnover. Comparison of the percent distribution of RBC sub-fractions in patients with Fontan circulation showed a trend for slightly shifted ratios towards younger RBC in comparison to previously published data from healthy controls [35]. Indicated higher RBC turnover might be relevant to meet demands, but further investigations are needed to address this particular issue.

In addition, we observed a corresponding significant increase in reticulocyte count after 24 h of hypoxia exposure, although a greater time interval would have been expected for measurable effects [28,53]. Koistinen et al. found a significant rise in reticulocyte count only after 5 days of hypoxia exposure under comparable conditions in healthy individuals [53]. Possibly, the finding might be explained by an already stimulated erythropoiesis in normoxia, with probably faster adapting mechanisms at increased hypoxemia.

Other than RBC count, RBC deformability is an important property affecting flow resistance [11]. Increased deformability parameters were previously described for younger RBC (respective reticulocytes) [57,58,59]. Additionally, a previous study suggests higher RBC deformability in patients with Fontan circulation compared to healthy controls, which might be related to an increased ratio of younger RBC, as suggested above [27]. However, a correlation analysis of deformability and reticulocyte count did not show a significant relationship (see Figure S1 in supplements). In addition, a higher reticulocyte count did not change RBC subfraction distribution and deformability at T2, as an increase in reticulocyte count of 10% may have been too small to affect total RBC fraction deformability parameters.

The influence of hypoxia on deformability is still controversial [16,27,31,32,60]. In our study, hypoxia did not affect deformability. The findings were in line with previous results in these patients [27], but also in healthy subjects, where RBC deformability did not significantly change at 2500 masl [16]. Additionally, deformability under an osmotic gradient remained unchanged. Taken together, these results might indicate stable RBC and flow properties under hypoxic conditions with adequate RBC hypoxia tolerance in patients with Fontan circulation.

Several studies have demonstrated that NO positively affects RBC deformability [18,19,46,61]. Connes et al. showed a dose-dependent effect with low concentrations leading to improvements, while high concentrations impaired RBC deformability [19]. In addition to this, Grau et al. demonstrated increased deformability because of NO-induced S-nitrosylation of the cytoskeleton protein ‘spectrin’ [46]. During hypoxia, the limited oxygen availability might result in decreased RBC-NOS activation, and reduced NO concentration could conceivably contribute to impaired deformability, as has been recently shown [18,61]. Nevertheless, these impairments occurred only at altitudes of 4000 m or higher [16,31]. The current results showed unaffected RBC-NOS activation and RBC nitrite/RSNO/Fe-NO values, indicating no relevant effect of the conducted hypoxic conditions on these parameters in the tested patient cohort. Furthermore, these results match the findings of the study conducted by our laboratory, where 2 h of hypoxia exposure also did not affect NO metabolism and RBC deformability in patients with Fontan circulation [27]. These results also match those of Grau et al., that determined unaltered RBC nitrite levels at 2500 masl in healthy subjects. Reductions in NO concentration and impairment in RBC deformability during hypoxia might be more likely to be the result of severe hypoxia with oxygen concentrations of 11 % or lower [16,27,31].

Furthermore, RBC ATP has been shown to affect deformability, due to energy-dependent maintenance of ionic and structural homeostasis within RBC [11,15,62]. Since RBC are denucleated cells, they can only regenerate ATP via glycolysis by two competing pathways: (1) The Embden-Meyerhof pathway and (2) the hexose monophosphate pathway. Metabolite flux depends on Hb conformation (oxygenated or deoxygenated) and cdB3 phosphorylation [62]. The Embden-Meyerhof pathway dominates under hypoxic conditions and mainly promotes ATP, whereas the hexose monophosphate pathway during full Hb-oxygenation mainly promotes nicotinamide adenine dinucleotide phosphate (NADPH) recycling for maintenance of antioxidant systems [11,62]. In our study, we observed no correlation between deformability and RBC ATP, suggesting sufficient RBC ATP levels in normoxia and in hypoxia for maintaining ionic balance and water content, as negative effects on deformability were only described for significantly reduced ATP levels [15].

As mentioned, ATP plays an important role in maintaining antioxidant systems, whereas hypoxia reduces antioxidant capacity by increasing oxidative stress [63,64]. In turn, a linear relationship was shown for oxidative stress and decrease of RBC deformability [65]. The age-dependent decrease in deformability in RBC has also mainly been attributed to oxidative stress [17]. Free oxidative radicals have been shown to impair deformability by damaging membrane and cytoskeletal proteins as well as lipids [17]. In addition, oxidative stress can initiate processes leading to reduced deformability, such as inhibition of Ca-ATPase, which is responsible for limiting the intracellular concentration of calcium [66,67]. Calcium, in turn, activates a cation channel that leads to leakage of potassium and disruption of cation homeostasis, which in turn leads to shrinkage of the cell and impairs deformability [17].

Hypoxia, independent of exposure time, appears to increase ROS/RNS. Although the underlying mechanisms are still not fully understood, previous studies implicated reduced mitochondrial redox potential, increased catecholamine production, and xanthine oxidase pathway activation as reasons for the development of oxidative stress [63,64,68,69]. To avoid potential cell damage by free oxygen species, RBC have a distinctive antioxidant system, consisting of enzymatic and non-enzymatic proteins, such as glutathione, catalase, glutathione peroxidase, and superoxide dismutase [17].

In our study, we demonstrated significantly increased ROS/RNS due to hypoxia, accompanied with a significantly decreased TAC. The finding is in line with the recently mentioned studies. However, RBC deformability was not affected by oxidative stress in the present investigation. This result is underlined by the unchanged nitrotyrosine concentration, an indicator of lipid peroxidation and cell damage due to oxidative stress [70]. The combination of unchanged ATP and nitrotyrosine concentrations with reduced TAC and unchanged RBC deformability, indicates an adequate antioxidant capacity of RBC, so that developing ROS/RNS can be sufficiently neutralized during hypoxia exposure; at least under the conditions applied herein.

In summary, our study suggests that 24 h of hypoxia exposure, akin to hypoxia encountered during airplane travel, does not lead to clinically relevant changes in hematological parameters and RBC deformability in patients with Fontan circulation. RBC TAC and RBC ATP might be adequate in maintaining cell homeostasis in such a way that RBC function and structure are not perturbed. Nevertheless, patients with Fontan circulation showed extraordinarily high Hct, which might be further increased secondary to hypoxia-induced EPO release, stimulating erythropoiesis and higher fluid loss during longer stays at high altitude. It may be prudent to ensure constant hydration to minimize the risks associated with impaired hemorheology.

Finally, further studies should examine the role of increasing concentrations of ROS/RNS, since a negative impact on RBC function cannot be completely excluded, especially for longer stays in altitude. The mechanisms might be even more important for physical activity in hypoxia, as different studies have demonstrated further increases in oxidative stress and changes in RBC structure during exercise [64,71,72,73]. Since this investigation lacks a control group, the data do not allow for an interpretation as to whether the observed response to hypoxic conditions might be comparable or different from that of healthy subjects. The aim of our study was to assess whether a hypoxic exposure corresponding to 2500 masl might bear any risks associated to altered RBC deformability or hematological parameters in patients with Fontan circulation.

In addition, total blood viscosity, hemorheological and hematological parameters should be investigated in hypobaric hypoxia, as there have been several studies that have indicated slightly different effects in ‘real’ altitudes [74]. Nevertheless, normobaric hypoxia is used in many studies as it shows good approximation to reality, is cost effective, and provides maximum safety for patients.

## Figures and Tables

**Table 1:** General characteristics of participating patients with Fontan circulation.

**Age**	Years	24.5 [16.3, 38.8] *
**Gender**	f/m	9/9
**Years since Fontan Completion**		20.3 ± 5.98
**Congenital Heart Malformation**		
	Tricuspid Atresia (TA)	4
	Pulmonary Atresia (PA)	1
	Pulmonary Atresia with ccTGA	2
	Hypoplastic Left Heart Syndrome (HLHS)	2
	Double Inlet Left ventricle (DILV)	5
	Double Outlet Right Ventricle (DORV)	2
	Double Inlet Right Ventricle (DIRV)	1
	Single Ventricle with ccTGA (SV)	1
**Functional Ventricle**		
	Left	10
	Right	8
**Anticoagulation**		
	Aspirin	6
	Phenprocoumon	8
	Warfarin	1
	New Oral Anticoagulants	2
	No Anticoagulation	1

* Median [min, max].

**Table 2:** SpO_2_ and RBC parameters in normoxia (T1), after 24 h in hypoxia (T2), and 60 min after leaving the altitude room in normoxia (T3).

			T1	T2	T3	Significance
						(*p*-Value)
SpO_2_		%	94 ± 3	87 ± 4	95 ± 4	<0.0001 ****^,††††^
pH (*n = 15*)			7.39 ± 0.03	7.4 ± 0.02	7.38 ± 0.03	0.008 **, 0.019 ^†^
Red blood cells		T/L	5.41 ± 0.63	5.57 ±0.77	5.5 ± 0.64	ns
Reticulocytes (n = 11)		G/L	74.86 ± 30.35	82.85 ± 34.07		0.005 **
Hemoglobin		g/dL	16.84 ± 2.01	17.66 ± 2.58	17.03 ± 2.33	ns
Hematocrit		%	47.89 ± 4.94	49.33 ± 6.55	48.77 ± 5.62	ns
MCV		fl	88.75 ± 3.33	88.63 ± 3.56	88.66 ± 3.27	ns
MCH		pg	30.91 ± 2.11	31.75 ± 2.67	30.91 ± 1.83	ns
MCHC		g/dL	34.81 ± 1.78	35.76 ± 2.23	34.86 ± 1.35	ns
RDW		%	13.54 ± 0.82	13.75 ± 0.89	13.69 ± 0.82	0.018 *
EPO_plasma_		mIU/mL	13.06 ± 2.82	30.95 ± 15.4	31.68 ± 14.19	<0.0001 ****^,‡‡‡‡^
RBC ATP		ng/µL	215.19 ± 29.89	203.53 ± 42.69	235.67 ± 47.5	ns

(*) symbolizing differences between T1 and T2, (‡) demonstrating differences between T1 and T3, and (†) showing differences between T2 and T3.

## Data Availability

The data supporting the findings of this study are available on reasonable request from the corresponding author J.A.H. The data are not publicly available due to restrictions described in the signed patient information letter ensuring that individual data will not be presented publicly in order to ensure the privacy of research participants.

## Supplementary Materials

The following supporting information can be downloaded at: https://www.mdpi.com/article/10.3390/metabo12111025/s1, Figure S1: Correlation of Δ reticulocyte count and Δ SS_1/2_:EI_max_ ratio (n = 9).
