# Supplementary figures and images for: Influence of 24 h Simulated Altitude on Red Blood Cell Deformability and Hematological Parameters in Patients with Fontan Circulation
Source: Metabolites. 2022 Oct 26;12(11):1025. doi: 10.3390/metabo12111025 (PMC9694701; doi:10.3390/metabo12111025)

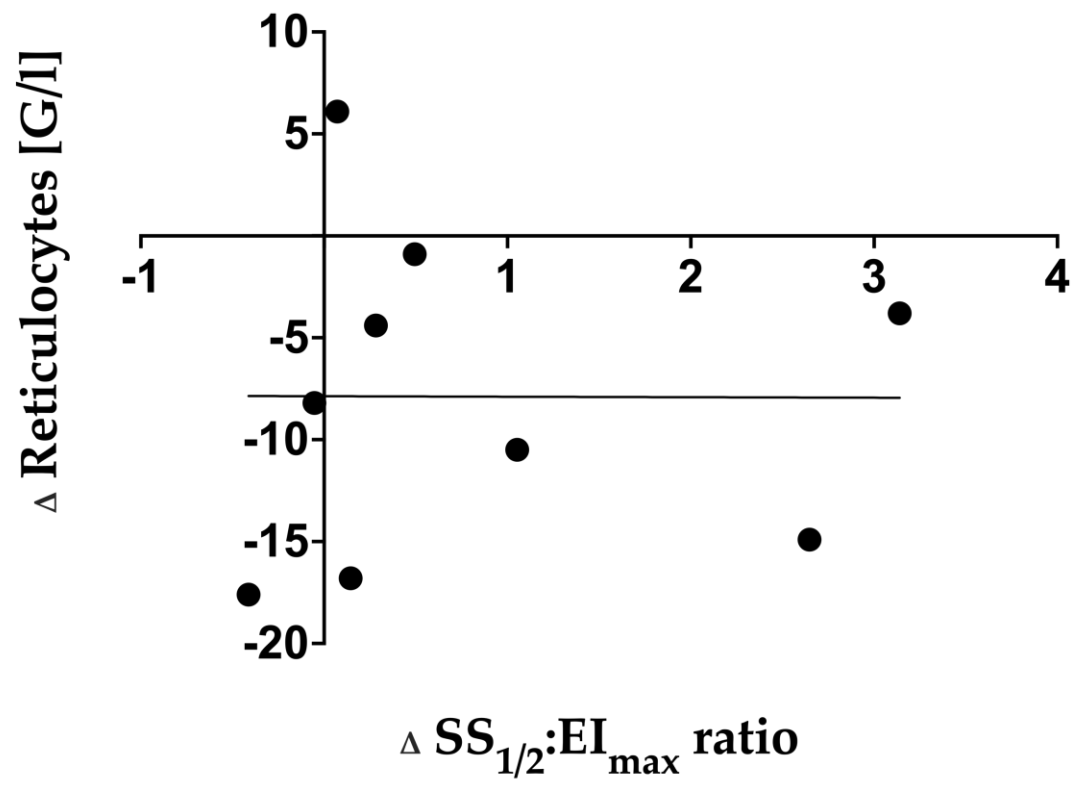

**Figure S1.** Correlation of  $\Delta$  reticulocyte count and  $\Delta SS_{1/2}:EI_{\max}$  ratio (n = 9).

Supplement: Supplementary file 1 [file metabolites-12-01025-s001.zip › metabolites-1914121-supplementary.pdf]
